# Effects of a polysaccharide-based multi-ingredient supplement on salivary immunity in non-elite marathon runners

**DOI:** 10.1186/s12970-019-0281-z

**Published:** 2019-03-25

**Authors:** Emma Roca, Elisabet Cantó, Lexa Nescolarde, Lidia Perea, Antoni Bayes-Genis, Oriol Sibila, Silvia Vidal

**Affiliations:** 1 Research Centre for Biomedical Engineering, Universitat Politècnica de Catalunya, Jordi Girona, 1-3, 08034 Barcelona, Spain; 2 Laboratory of Experimental Immunology, Biomedical Research Institute Sant Pau (IIB Sant Pau), Hospital de la Santa Creu i Sant Pau, Barcelona, Catalonia Spain; 3 Department of Electronic, Universitat Politècnica de Catalunya, Barcelona, Spain; 4 Department of Cardiology, Hospital Universitari Germans Trias i Pujol, Badalona, Spain; 5 Department of Medicine, Universitat Autònoma de Barcelona, Barcelona, Spain; 6 Research Program, Fundació Institut d’Investigació en Ciències de la Salut Germans Trias i Pujol, Badalona, Spain; 7 Respiratory Department, Hospital de la Santa Creu i Sant Pau, Autonomous University of Barcelona and Biomedical Research Institute Sant Pau (IIB Sant Pau), Barcelona, Catalonia Spain

**Keywords:** Glycans, Dietary plant-derived polydisperse polysaccharide supplementation, Salivary sIgA, Inflammatory chemokines, Marathon, Strenuous exercise

## Abstract

**Background:**

Extreme exercise may alter the innate immune system. Glycans are involved in several biological processes including immune system regulation. However, limited data regarding the impact of glycan supplementation on immunological parameters after strenuous exercise are available. We aimed to determine the impact of a standardized polysaccharide-based multi-ingredient supplement, Advanced Ambrotose© complex powder (AA) on salivary secretory Immunoglobulin A (sIgA) and pro- and anti-inflammatory protein levels before and after a marathon in non-elite runners.

**Methods:**

Forty-one male marathon runners who completed the 42.195 km of the 2016 Barcelona marathon were randomly assigned to two study groups. Of them, *n* = 20 (48%) received the AA supplement for 15 days prior the race (AA group) and *n* = 21 (52%) did not receive any AA supplement (non-AA group). Saliva and blood samples were collected the day before the marathon and two days after the end of the race. Salivary IgA, pro-inflammatory chemokines (Gro-alpha, Gro-beta, MCP-1) and anti-inflammatory proteins (Angiogenin, ACRP, Siglec 5) were determined using commercially ELISA kits in saliva supernatant. Biochemical parameters, including C-reactive protein, cardiac biomarkers, and blood hemogram were also evaluated.

**Results:**

Marathon runners who did not receive the AA supplement experienced a decrease of salivary sIgA and pro-inflammatory chemokines (Gro-alpha and Gro-beta) after the race, while runners with AA supplementation showed lower levels of anti-inflammatory chemokines (Angiogenin). Gro-alpha and Gro-beta salivary levels were lower before the race in the AA group and correlated with blood leukocytes and platelets.

**Conclusions:**

Changes in salivary sIgA and inflammatory chemokines, especially Gro-alfa and Gro-beta, were observed in marathon runners supplemented with AA prior to the race. These findings suggested that AA may have a positive effect on immune response after a strenuous exercise.

## Background

In recent years, there has been a significant increase of participants in ultra-endurance races such as marathons and ultramarathons. In the United States alone, marathon runners have increased from 25,000 in 1976 to almost 503,000 in 2010 [18]. Competing in very strenuous events imposes severe metabolic stress and causes acute responses that may negatively alter the immune system [17]. A high level of physical demand during such events induces a wide range of metabolic changes and causes micro-injuries in the muscles and other tissues. This physical demand increases the migration of white blood cells to the sites of injury, and induces acute phase inflammatory reactions [5, 16, 28]. The local response to tissue injury involves the production of a large number of acute phase proteins and cytokines related to innate immunity such as secretory Immunoglobulin A (sIgA) or inflammatory chemokines [9, 30]. Secretory Immunoglobulin A (sIgA) is a first line of defense against external agents, serving as a noninvasive biomarker of mucosal immunity [10]. Several studies have shown significant sIgA changes following strenuous exercise [3, 19, 24]. Other important immune factors present in mucosal secretion also change after prolonged running [6, 20, 24]. These changes include: pro-inflammatory chemokines, such as Gro-alpha (neutrophil-activating protein 3 and melanoma growth stimulating activity), Gro-beta (macrophage inflammatory protein, secreted by monocytes and macrophages and chemotactic for polymorphonuclear leukocytes and hematopoietic stem cells) and MCP-1 (to recruit monocytes, memory T cells, and dendritic cells to the sites of inflammation produced by either tissue injury or infection); anti-inflammatory proteins such as Angiogenin (associated with altered normality through angiogenesis and through activating gene expression that suppresses apoptosis), Adipokine ACRP30 (cell signaling protein secreted by adipose tissue involved in regulating glucose levels as well as fatty acid breakdown) and Siglec5 (Sialic acid-binding Ig-like lectin 5).

Glycans (a generic term for any sugar or assembly of sugars, in free form or attached to another molecule) are directly involved in physiology [31]. Glycans are involved in inflammation and immune system activation [11]. A standardized polysaccharide-based multi-ingredient supplement including glycans (Advanced Ambrotose© complex powder (AA)) may produce a significant overall shift towards an increased sialylation of serum glycoproteins. Sialylation changes can have a key role in many aspects of the immune response [2]. It is therefore not surprising that one of the physiological effects of these polysaccharides is the immunomodulation through the glycosylation of proteins [15]. AA is a saccharide supplement containing a standardized combination of plant polysaccharides (a source of mannose, galactose, fucose, xylose, glucose, n-acetyl-glucosamine, n-acetyl-neuraminic acid, and n-acetyl-galactosamine) that may regulate immunity. In controlled human trials, polysaccharide intake enhanced the immune system biomarkers in the blood of healthy adults [22]. The effect of dietary supplements with polysaccharides on retired professional football players supported and optimized their quality of life [25]. However, no data regarding the impact of AA supplementation on healthy marathon runners performing strenuous physical activity is available.

Our hypothesis is that glycan supplementations before strenuous physical activity enhances immune function and balances pro-inflammatory and anti-inflammatory proteins. Therefore, the aim of this study is to determine the impact of AA in the levels of sIgA, pro-inflammatory chemokines and anti-inflammatory proteins before and after running a marathon in non-elite marathoners.

## Methods

### Participants

This study is a part of the SUMMIT project (Health in Ultra-Marathons and their Limits), whose objective is to evaluate the behavior of certain clinical parameters in different races and was approved by an institutional review board (IIBSP-SUMMIT-2016-02). In this study, 41 male non-elite runners of the Barcelona Marathon 2016 participated and they gave signed informed consent.

All runners were weighed on race morning within 2 h of race start in racing attire with running shoes and immediately after completing the race using the same scale Jata 565 model. Later the body mass index (BMI) was calculated using the formula BMI = weight (kg) / height (m^2^). The mean age was 39.3 ± 9.2 years old.

### Study groups

Twenty participants (48%) received AA supplementation prior to the race (AA group), while 21 participants (52%) did not receive the AA supplement prior to the race (non-AA group). AA was administrated at dose of 8 g/day (with Arabinolactan, *Aloe Vera* extract, rice starch, ghatti gum, gum Tragacanth, Glucosamine HCl, Wakame algae extract) for 15 days before the marathon. The 41 athletes were selected for being homogeneous in age, weekly training hours, years of training, weight and height (Table 1), and were randomly assigned into the two study groups.

**Table 1:** Participant characteristics, anthropometric and training data before the marathon

Baseline characteristics	Total cohort	Non-AA group	AA group	*p*-value
Number of runners	41	21	20	
Age (years)	39.3 ± 9.2	39.3 ± 12.8	39.2 ± 4.9	0.717
Weight (Kg)	77.0 ± 11.0	74.0 ± 12.0	78.5 ± 12.0	0.208
Height (cm)	178.0 ± 8.0	178.0 ± 7.0	179.5 ± 8.0	0.142
BMI (kg/m^2^)	24.1 ± 3.4	23.8 ± 3.0	24.2 ± 3.7	0.696
Training (years)	8.0 ± 8.5	7.0 ± 6.5	9.0 ± 12.3	0.537
Training (hours/week)	7.0 ± 5.3	6.0 ± 4.5	7.0 ± 4.9	0.106
Loss weight (%)	3.1 ± 1.8	3.1 ± 2.9	3.1 ± 1.7	0.276
Race time	3.5 ± 0.5	3.4 ± 0.6	3.6 ± 0.5	0.265

AA group: Participants received Advanced Ambrotose Complex Powder (AA) supplementation prior the race

Non-AA group: Participants did not received any AA supplement prior the race

*BMI*: body mass index

Values are reported as mean ± SD for the quantitative and in percentage for the categorical. ANOVA test was used for parametric variables; Kruskal Wallis test was used for non parametric variables; Chi-Square Pearson for categorical variables

### Saliva and blood collection

Saliva samples were collected 48 h before the marathon (d0 samples) and two days after the end of the race (d2 samples). Saliva was collected into clean, sterile tubes, maintained at 4 °C before centrifugation at 9500×g 10 min at 4 °C. Supernatant was aspirated and 3 different aliquots were frozen at − 20 °C, until evaluated.

Three 10 mL blood samples were obtained from the antecubital vein 48 h before the marathon (d0 samples), at completion (d1 samples), and 48 h after the race (d2 samples). After the marathon, samples (d1) and weights were obtained within the 10 min interval after completing the race and before drinking any fluid or emptying the bladder.

### Saliva analysis

Saliva samples were thawed and total saliva protein was quantified using Qubit protein Assay Kit (molecular probes Life Technologies). Salivary IgA (Human IgA, Platinum ELISA, ebioscience, Affymetrix, Santa Clare, CA), Lactoferrin, Lysozyme (AssayPro, St, Charles, MO, USA), Gro-alpha/CXCL1, Gro-beta/CXCL2 and MCP-1 (Elabscience, Houston, Texas) were measured by ELISA according to manufacturer’s instructions. Limits of detection were for IgA: 1.6 ng/ml; lactoferrin: 0.625 ng/ml; lysozyme: 0.0781 ng/ml; Gro-alpha/CXCL1: 15.63 pg/ml, Gro-beta/CXCL2: 15.63 pg/ml, MCP-1: 15.63 pg/ml, Angiogenin: 1.64 pg/ml, ARCP30: 24.69 pg/ml and Siglec 5: 6.86 pg/ml. The data was expressed as concentration of each protein relative to total saliva protein concentration.

### Blood analysis

The complete blood counts were performed on the Unicel DxH800 automated hematology analyzer (Beckman Coulter, Miami, FL, USA). N-terminal pro-B-type natriuretic peptide (NT-proBNP) was quantified in whole EDTA blood using the AQT90 FLEX immunoassay (Radiometer Medical, Copenhagen, Denmark). Serum creatine kinase (CK) and C-reactive protein (CRP) were determined using the AU-5800 Chemistry Analyzer (Beckman Coulter). Troponin T was measured from serum, using a High Sensitive Troponin-T assay in a Cobas e601 platform (Roche Diagnostics, Barcelona, Spain). ST2 was measured from serum samples using a high-sensitivity sandwich monoclonal immunoassay (Pressage® ST2 assay, Critical Diagnostics, San Diego, CA, USA).

### Statistical analysis

The Kolmogorov-Smirnov test was applied to test the normal distribution of the data. All variables, with a normal distribution, were reported as mean ± standard deviation (SD). The rest of the variables were reported as median (interquartile rank) (IQR). T-test and paired t-test were respectively used for the comparison of independent and related variables with normal distribution. Wilcoxon test was used for the comparison of related variables with non-normally distributed data. Pearson’s and Spearman’s coefficients were respectively used to correlate changes between normal and between non-normal distributed variables. ANOVA and Kruskal-Wallis test were respectively used for comparative analyses of multiple normal and non-normal distributed data. Chi-square tests were used for the comparison of frequencies. *P* values less than 0.05 were considered significant.

## Results

### Study participants

Forty-one male non-elite marathon runners completed the 42.195 km of the 2016 Barcelona Marathon. Mean finishing time was 3 h and 28 min ± 0. 41 h. Median (interquartile range) age was 39 ± 9 years old, with a weight of 77 ± 11 kg, height of 178 ± 8 cm and Body Mass Index (BMI) of 24.1 ± 3.4. Median training years were 8 ± 8, with a median of 7 ± 5 h per week.

No significant differences in age, weight, height, and training were found between runners who received AA supplementation and those who did not (Table 1).

### Plasma and blood measurements

C-reactive protein increased significantly 48 h after the marathon (d2), although no significant differences between AA and non-AA groups were found. Similarly, all evaluated cardiac (Hs-TnT, St2 and NTproBNP) and muscle damage (CK) biomarkers increased after the race, but no significant differences between participants in either group were found (Table 2).

**Table 2:** Plasma and blood measurements before, after and 48 h after the marathon in 41 runners

Biochemical values	Total cohort	Non-AA group	AA group	*p*-value
CRP_d0 (mg/dL)	0.85 ± 1.18	0.60 ± 1.28	1.00 ± 1.23	0.683
CRP_d1 (mg/dL)	0.55 ± 0.85	0.45 ± 2.91	0.95 ± 2.48	0.606
CRP_d2 (mg/dL)	7.6 ± 4.65	6.80 ± 5.00	8.95 ± 4.25	0.359
CK_d0 (U/L)	170.5 ± 123.8	188.5 ± 172.3	136.0 ± 125.3	0.633
CK_d1 (U/L)	566.0 ± 282.3	566.0 ± 251.5	567.5 ± 396.3	0.958
CK_d2 (U/L)	764.5 ± 540.8	788.5 ± 787.8	650.0 ± 513.8	0.368
Hs-TnT_d0 (ng/L)	3.1 ± 2.7	2.9 ± 2.0	3.4 ± 5.0	0.407
Hs-TnT_d1 (ng/L)	49.6 ± 68.1	48.9 ± 68.8	49.9 ± 68.8	0.449
Hs-TnT_d2 (ng/L)	4.8 ± 6.3	4.9 ± 7.2	4.6 ± 3.7	0.334
St2_d0 (ng/mL)	34.0 ± 14.8	34.7 ± 16.2	28.6 ± 16.0	0.394
St2_d1 (ng/mL)	54.9 ± 30.3	59.0 ± 40.5	43.3 ± 30.4	0.256
St2_d2 (ng/mL)	32.9 ± 20.5	37.7 ± 19.3	29.4 ± 17.1	0.059
NTproBNP_d0 (ng/L)	70.0 ± 0	70.0 ± 0	70.0 ± 0	0.336
NTproBNP_d1 (ng/L)	94.5 ± 70.5	100.5 ± 50.3	85.5 ± 221.3	0.392
NTproBNP_d2 (ng/L)	70.0 ± 0	70.0 ± 0	70.0 ± 0.5	0.845
Hemoglobin_d0 (g/L)	14.8 ± 1.3	14.8 ± 1.1	14.7 ± 1.6	0.350
Hemoglobin_d1 (g/L)	14.7 ± 1.1	14.8 ± 1.0	14.5 ± 0.3	0.173
Hemoglobin_d2 (g/L)	14.3 ± 0.7	14.4 ± 0.7	14.2 ± 0.9	0.170
Hematocrit_d0	43.7 ± 3.0	43.7 ± 3.1	43.8 ± 3.1	0.327
Hematocrit_d1	44.0 ± 4.2	44.3 ± 3.2	43.3 ± 3.4	0.132
Hematocrit_d2	42.7 ± 2.2	42.8 ± 1.8	42.2 ± 3.7	0.114
Erythrocytes_d0 (10Exp12/L)	4.9 ± 0.6	4.9 ± 0.4	5.1 ± 0.7	0.903
Erythrocytes_d1 (10Exp12/L)	5.0 ± 0.5	5.0 ± 0.4	5.0 ± 0.8	0.586
Erythrocytes_d2 (10Exp12/L)	4.8 ± 0.4	4.8 ± 0.3	4.9 ± 0.5	0.532
Platelets_d0 (10Exp9/L)	0.2 ± 0.0	0.2 ± 0.0	0.2 ± 0.0	0.063
Platelets_d1 (10Exp9/L)	0.2 ± 0.0	0.2 ± 0.1	0.2 ± 0.0	0.852
Platelets_d2 (10Exp9/L)	0.2 ± 0.0	0.2 ± 0.0	0.2 ± 0.0	0.555
Leukocyte_d0 (10^9^/L)	6.5 ± 1.9	6.4 ± 1.3	6.5 ± 3.2	0.860
Leukocyte_d1 (10^9^/L)	12.2 ± 5.3	16.1 ± 4.4	13.2 ± 4.6	0.032
Leukocyte_d2 (10^9^/L)	6.6 ± 1.3	6.6 ± 1.4	6.6 ± 1.8	0.217

AA group: Participants received Advanced Ambrotose Complex Powder (AA) supplementation prior the race

Non-AA group: Participants did not received any AA supplement prior the race

*CK* creatine kinase, *CRP* C-reactive protein, *HGB* hemoglobin, *Hs-TnT* high-sensitivity troponin T, *NT-proBNP* N-terminal pro-B-type natriuretic peptide

Values are reported as mean ± SD for the quantitative and in percentage for the categorical. ANOVA test was used for parametric variables; Kruskal Wallis test was used for non parametric variables; Chi-Square Pearson for categorical variables

Hemoglobin, hematocrit, erythrocytes and platelets counts did not change after the race (d1), with no differences between groups. However, a marked increase in leukocyte counts per L was found in both groups after the race (d1) and participants who received AA supplementation for 15 days prior to the race experienced a lower increase compared to those without the AA supplementation (13.2 ± 4.6 vs 16.1 ± 4.4 × 10^9^/L, *p* = 0.03) (Table 2).

### Salivary IgA

**Fig. 1:**
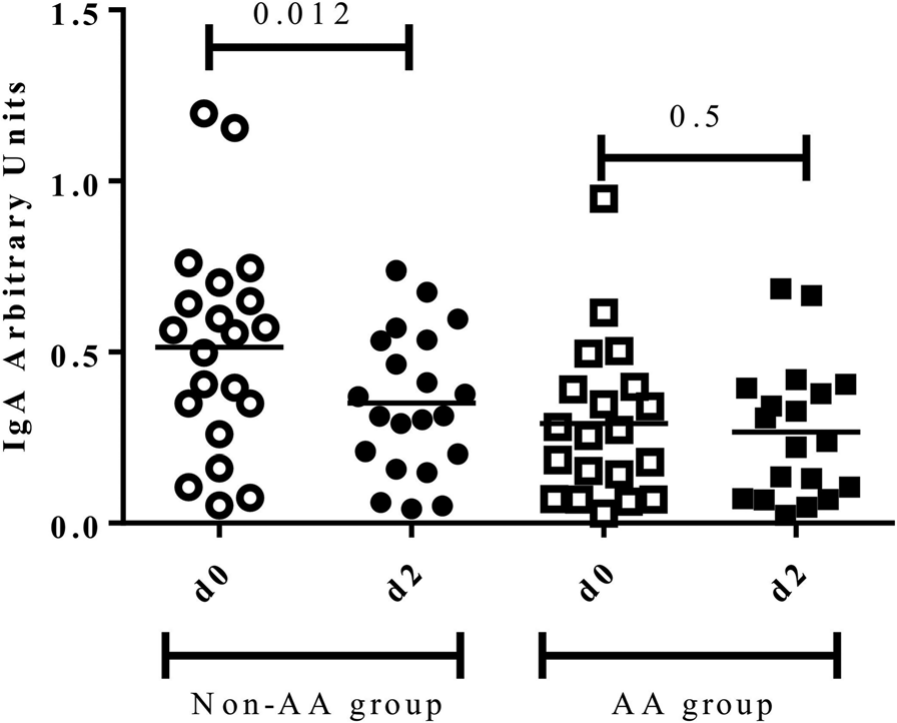
Salivary IgA levels before (d0) and 48 h after the marathon (d2) between groups

No differences in salivary IgA levels normalized to total salivary protein before (d0) and 48 h after the race (d2) were found (0.47 ± 0.44 vs 0.37 ± 0.29, *p* = 0.6). However, when participants with and without AA supplementation were compared, a decrease of sIgA 48 h after the race (d2) was observed in the non-AA group (0.55 ± 0.37 vs 0.31 ± 0.35, *p* = 0.01), while no differences before (d0) and 48 h after the race (d2) in the AA group were found (0.25 ± 0.33 vs 0.24 ± 0.32, *p* = 0.5) (Fig. 1).

### Salivary pro inflammatory proteins

**Fig. 2:**
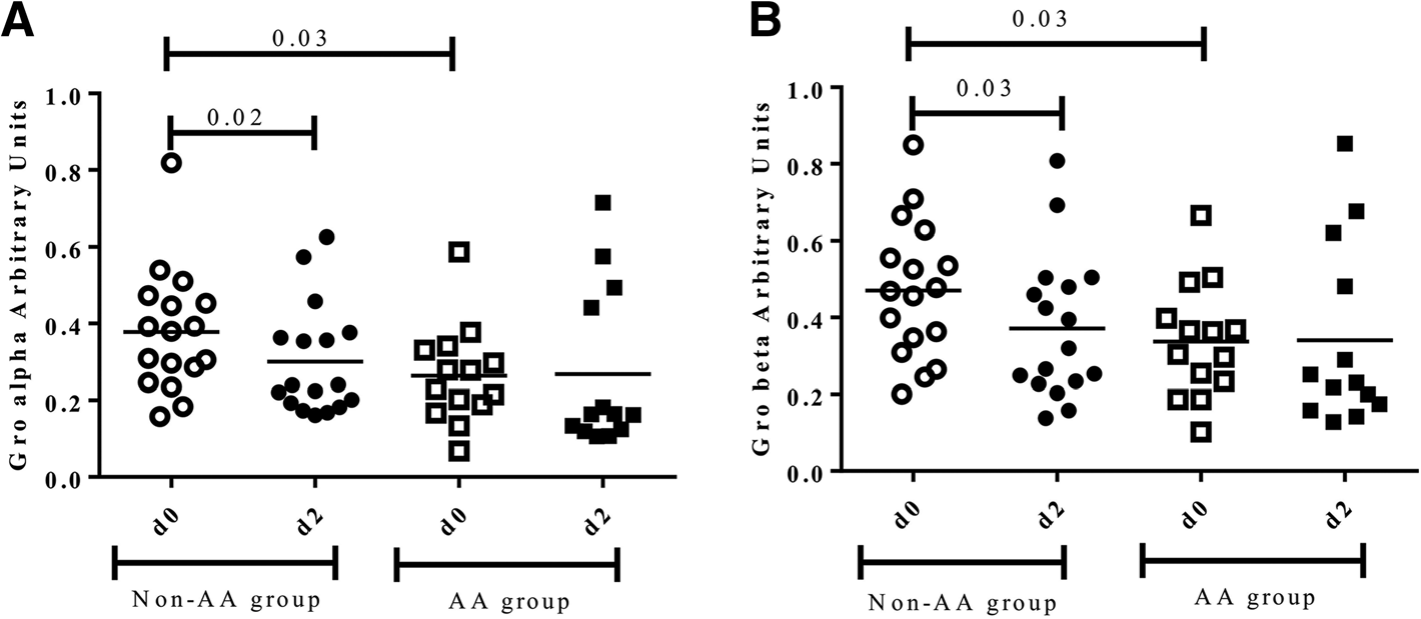
Salivary pro inflammatory proteins, gro alpha (**a**) and gro beta (**b**) before (d0) and 48 h after the marathon (d2) between groups

A decrease of pro-inflammatory salivary proteins such as Gro-alfa, Gro-beta and MCP-1 after the race was observed in all participants, although differences were not statistically significant (data not shown). Nevertheless, the decline in Gro-alpha (Fig. 2a) and Gro-beta (Fig. 2b) was statistically significant in those runners who did not receive the AA supplement (0.38 ± 0.20 vs 0.24 ± 0.18, *p* = 0.02 and 0.47 ± 0.26 vs 0.32 ± 0.26, *p* = 0.03), while differences were not observed in the AA group. Furthermore, basal levels of Gro-alpha and Gro-beta were significantly lower (p = 0.03) in the AA group at baseline before the marathon (d0) compared to the non-AA group (Fig. 2).

### Salivary anti-inflammatory proteins

**Fig. 3:**
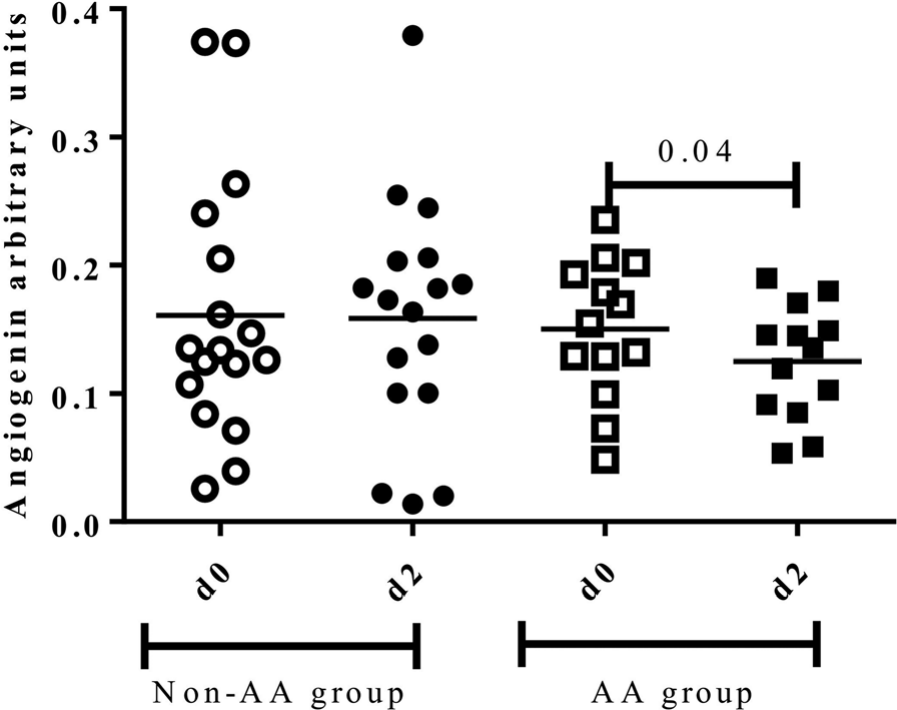
Salivary anti-inflammatory protein Angiogenin before (d0) and 48 h after the marathon (d2) between groups

No statistically significant differences of salivary anti-inflammatory proteins such as ACRP, angiotensin, and Siglec 5 were observed before (d0) and 48 h after the marathon (d2) (data not shown). However, in those runners of the AA group, a significant decrease of Angiogenin 48 h after the race (d2) was observed (0.18 ± 0.08 vs 0.14 ± 0.07, *p* = 0.04), while differences were not observed in the non-AA group (0.3 ± 0.13 vs 0.17 ± 0.10, *p* = 0.7) (Fig. 3).

### Systemic correlations

**Fig. 4:**
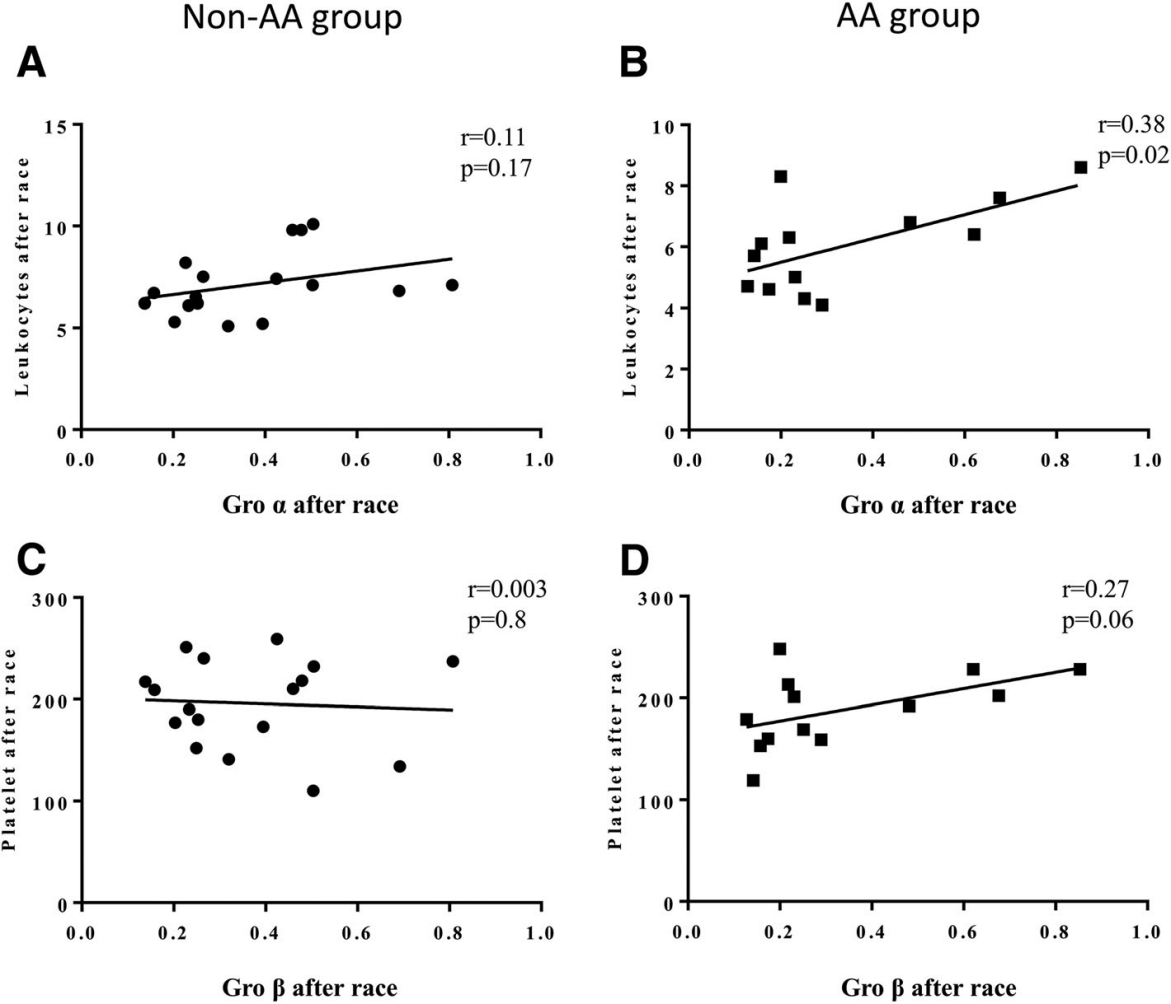
Systemic correlations 48 h after the marathon (d2) between: salivary Gro-alpha levels with leukocyte counts in non-AA group (**a**) and AA group (**b**) and Gro-beta salivary levels and blood platelet counts in non-AA group (**c**) and AA group (**d**)

After the race, those runners who received the AA supplement (AA group) showed positive correlations between salivary Gro-alpha levels with leukocyte counts per L (r = 0.38, *p* = 0.02) and a tendency between Gro-beta salivary levels and blood platelet counts (r = 0.27, *p* = 0.06). These systemic correlations were not observed in the non-AA group (Fig. 4).

## Discussion

In our study, we demonstrated significant changes in salivary biomarkers of immune function in healthy, non-elite athletes before and after a strenuous exercise like an asphalt marathon, after consuming a polysaccharide-based multi-ingredient supplement, AA, for 15 days prior to exercise. Specifically, a decrease in salivary sIgA, Gro-alfa and Gro-beta were observed after the marathon in those runners who did not receive AA supplement prior the race, while those runners who received the AA supplement showed a decrease in a salivary Angiogenin. Gro-alpha and Gro-beta salivary levels were lower before the race in the AA group and correlated with the counts of blood leukocytes and platelets. These findings suggest that AA supplementation produces changes in salivary immunity that may have a positive effect on immunity before and after a marathon.

sIgA is a key component of innate immunity and provides a first line of defense against pathogens at mucosal surfaces [8]. Numerous studies have assessed the saliva sIgA response to prolonged strenuous exercise to explore whether immunity may be temporarily compromised after the exercise. Most of the studies described a post-exercise decrease in sIgA, including Nordic skiers after a 50 km race [29] or trained cyclists cycling for more than 2 h on a stationary ergometer [32]. In marathon runners, a significant decrease of sIgA following a marathon race has been described [19], which was independent of gender, age or carbohydrate ingestion. In our study, runners with AA supplementation did not experience a significant decrease in sIgA after the marathon, which was observed in runners without the AA. These findings suggested that AA supplement enhanced immunity by avoiding the sIgA decrease after the race. Different studies have postulated that glycosylation plays an important role in the biosynthesis and biological activity of the proteins involved in antigen recognition, such as sIgA [23]. Further studies are needed to explore this pathway, which may be crucial in order to better understand the effect of strenuous exercise on mucosal immunity.

Supplementation with glycans can also result in salivary glycol-modifications of proteins. These modifications can regulate the synthesis and/or degradation of pro- and anti-inflammatory molecules participating in the immune response of runners to strenuous effort. Several inflammatory chemokines have demonstrated a potential role in the regulation of immune response during exercise [27]. In our study, we demonstrated that Gro-alfa and Gro-beta, two pro-inflammatory chemokines, involved in the attraction of neutrophils to the site of inflammation, decreased in saliva after the race in those runners who did not receive AA. It has been described that moderate exercise suppressed Gro-alfa [4] and, in experimental models, exercise down-regulates multiple inflammatory cytokines [26], including Gro-alfa and Gro-beta, that results in a systemic anti-inflammatory effect [1, 13]. These findings were not observed in runners who received the AA supplement, suggesting that they had better immunoregulation. Runners with AA supplementation have also showed a decrease in salivary Angiogenin levels, which was not detected in runners without AA. It suggests that salivary pro-inflammatory proteins can be buffered with anti-inflammatory ones, especially those produced by runners supplemented with AA. In addition, it is important to note that after 15 days on AA, the baseline levels of Gro-alfa, Gro-beta and Angiogenin were different in runners with and without AA. Interpretations of all these salivary inflammatory changes are complex and require further more detailed studies.

The systemic effect of glycans has been described in several studies [15]. Interestingly, it is postulated that glycans may regulate leukocyte activity [21]. In our study, runners who received the AA supplement experienced a lower increase in total blood leukocyte counts after the race compared to those runners without the AA supplement. Furthermore, a correlation between salivary levels of Gro-alpha with blood leukocyte counts and salivary Gro-beta levels with blood platelet counts were observed in the AA group. All in all, these findings suggested a relationship between salivary and systemic immunity, especially in those runners who received the AA supplement prior to the race. The association between high dietary protein during high-intensity training and reduction in respiratory symptoms in elite cyclists by restoring impairments in leukocyte trafficking has also been reported [35] The influence of ingredients other than glycans on the immune system has been reported. For example, brown seaweed [33], *Aloe vera* [12] and glucosamine [14] have antioxidant activity and anti-inflammatory effects. Arabinogalactan decreases the incidence of infectious episodes by improving serum-antigen specific IgG and IgE response to *Streptococcus pneumonia* [7]. Starch supplementation promotes the growth of commensal bacteria that may improve bowel health [34]. However, more work is needed to clarify the mechanism that may explain these important results.

Our study has limitations. First, we only have studied men, and we cannot exclude the effect of glycans in salivary immunity based on sex. Second, due to the small sample size of runners included, the results should be validated in further studies before generalizing them. Third, runners received the AA supplement for 15 days prior the race, and the effect of taking this supplement for a longer time may be different. Fourth, the influence of ingredients included in the AA supplements which are not glycans such as glucosamine and *Aloe Vera* on the immune system cannot be ruled out, and further studies in non-elite marathon runners should be performed to better clarify this important point.

## Conclusion

In conclusion, our study documented significant changes in salivary and systemic immunological biomarkers in marathon runners after consuming a polysaccharide-based multi-ingredient supplement AA, before and after the race. Further research using randomized, double-blind, placebo-controlled design will extend knowledge of the potential benefits nutritional dietary supplementation with polysaccharides may have in marathon runners.

## Abbreviations

AA: Standardized dietary plant-derived polydisperse polysaccharide Advanced Ambrotose© complex powder
BMI: Body mass index
CK: Serum creatine kinase
CRP: C-reactive protein
HGB: Hemoglobin
Hs-TnT: High-sensitivity troponin T
NT-proBNP: N-terminal pro-B-type natriuretic peptide
sIgA: Salivary secretory Immunoglobulin A
